# Responsive Industrial Polymers: A Marriage of Polyurethanes with Liquid Crystal Elastomers?

**DOI:** 10.1021/acsami.5c09198

**Published:** 2025-05-27

**Authors:** Lansong Yue, Xue Wan, Tankut Türel, Albert P. H. J. Schenning, Željko Tomović, Michael G. Debije

**Affiliations:** † Stimuli-Responsive Functional Materials and Devices, Department of Chemical Engineering and Chemistry, 3169 Eindhoven University of Technology, Eindhoven 5600 MB, The Netherlands; ‡ Polymer Performance Materials Group, Department of Chemical Engineering and Chemistry, Eindhoven University of Technology, Eindhoven 5600 MB, The Netherlands; § Interactive Polymer Materials (IPM), Eindhoven University of Technology, Eindhoven 5612 AE, The Netherlands; ∥ Institute for Complex Molecular Systems (ICMS), Eindhoven University of Technology, Eindhoven 5600 MB, The Netherlands

**Keywords:** liquid crystal elastomers, polyurethanes, stimuli-responsive actuators, smart materials, industrial polymers

## Abstract

Responsive polymers have yet to significantly impact the marketplace. In this Perspective, we offer a glimpse of a possible future industrial-scale responsive polymer. We begin by briefly reviewing two different existing polymer materials, one with high volume, excellent processability, and commercial impact (polyurethanes), the other with stimuli responsive functional properties (liquid crystal elastomers). We explore the possibilities of combining the properties of these two disparate entities into a single material. We offer intriguing possibilities for a bulk polymer with both responsivity and processability that could compete in the market with the long-established residents and discuss some of the research roadblocks that need to be overcome to reach this lofty goal.

## Graphical abstract

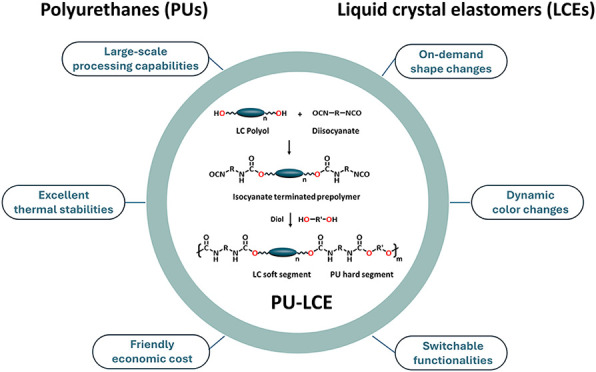

## 1 Introduction

Polymers have contributed enormously to the well-being of humanity, produced in enormous quantities and used in endless applications. However, the vast majority of bulk polymers employed today were all discovered at least 60 years ago.^1^ For example, polyurethanes (PUs), one of the most important polymer families, were first made in 1937.

With the maturation of the industry and increasing affluence of the international population, there has been a clamor for polymers with enhanced performance and environmental responsivity not available in classical materials. For example, polymers that react to changes in their environment with alterations in their shape and/or optical properties are the next generation: imagine footwear responding to temperature or humidity changes and appearing in different colors, color-change memory foam mattress to diagnose stress points, fibers for clothing that form a closer fit upon heating by the body, varnishes with responsive optical effects such as warning arrows appearing to direct people to safety when exposed to high levels of toxic gases, medical wrappings that compress or exuding medications when triggered by specific wavelengths of light, automotive morphing skins that adjust shape to reduce aerodynamic drag and interiors with personalized optical responses, to speculate upon just a few possibilities.

A considerable body of research has been produced studying the stimuli-responsive properties of liquid crystal elastomers (LCEs).^2–5^ LCEs combine the anisotropy of liquid crystals (LCs) in both optical and mechanical properties with the rubbery nature of elastomers. Despite the remarkable responsive properties of the LCE, demonstrating large shape and optical variations in response to changing external conditions, they have had no market impact as they are not appropriate for large-scale industrial production and processing.

We propose a radical new concept for stimuli-responsive polymers combining PUs and LCEs capable of being produced at large scales and processed by standard industrial technologies.^6^ While interpenetrating PU and LC acrylate elastomer networks with good physical properties have previously been studied,^7–9^ they have not been commercially exploited. We begin our Perspective by giving a bit of insight into the properties and applications of PUs and LCEs individually, discussing their advantages and disadvantages as materials. We then speculate on the possibilities of combining properties; that is, the responsivity and actuation capabilities of the LCE and the industrial scale processability of the PU, and suggest research that needs to be done that, if successful, could introduce to the world a new polymer family with responsivity, adapting to local environmental changes, bringing a disruptive new material to market.

## 2 Polyurethanes (PUs) in a Nutshell

Polymer-based materials, commonly referred to as “plastics”, are indispensable in the modern world, finding applications across virtually every aspect of daily life. Their exceptional properties—light weights, ease of processing, versatility, durability, adaptability, energy efficiency, and cost-effectiveness—make them unparalleled among materials.^10^ Global plastic production exceeded 400 million tons in 2022, with polyethylene (PE) and polypropylene (PP) being the most produced, followed by poly­(vinyl chloride) (PVC), PU, and poly­(ethylene terephthalate) (PET).^11^ Notably, global PU production reached 30 million tons in 2020,^12^ marking PU as one of the most important polymer families.

PUs were first developed by Dr. Otto Bayer and his team through fundamental diisocyanate-polyol polyaddition reactions, with the earliest example synthesized by reacting a diisocyanate with a polyester containing two hydroxyl end groups.^13–16^ PU polymers first found commercial use between 1945 and 1947 with applications in elastomers, coatings, and adhesives. These innovations were later followed by the introduction of flexible foams in 1953 and rigid foams in 1957, marking a series of advancements that reshaped industries worldwide.^15^ Since then, PUs have become indispensable across a wide range of applications and industries due to their unmatched versatility and unique combination of properties, including durability, flexibility, and lightweight nature.^17,18^ Today, flexible and rigid foams dominate PU applications,^15,17–19^ followed by adhesives,^20–22^ coatings,^23,24^ sealants,^25^ and elastomers^15,26,27^ (Figure 1).

**Figure 1:**
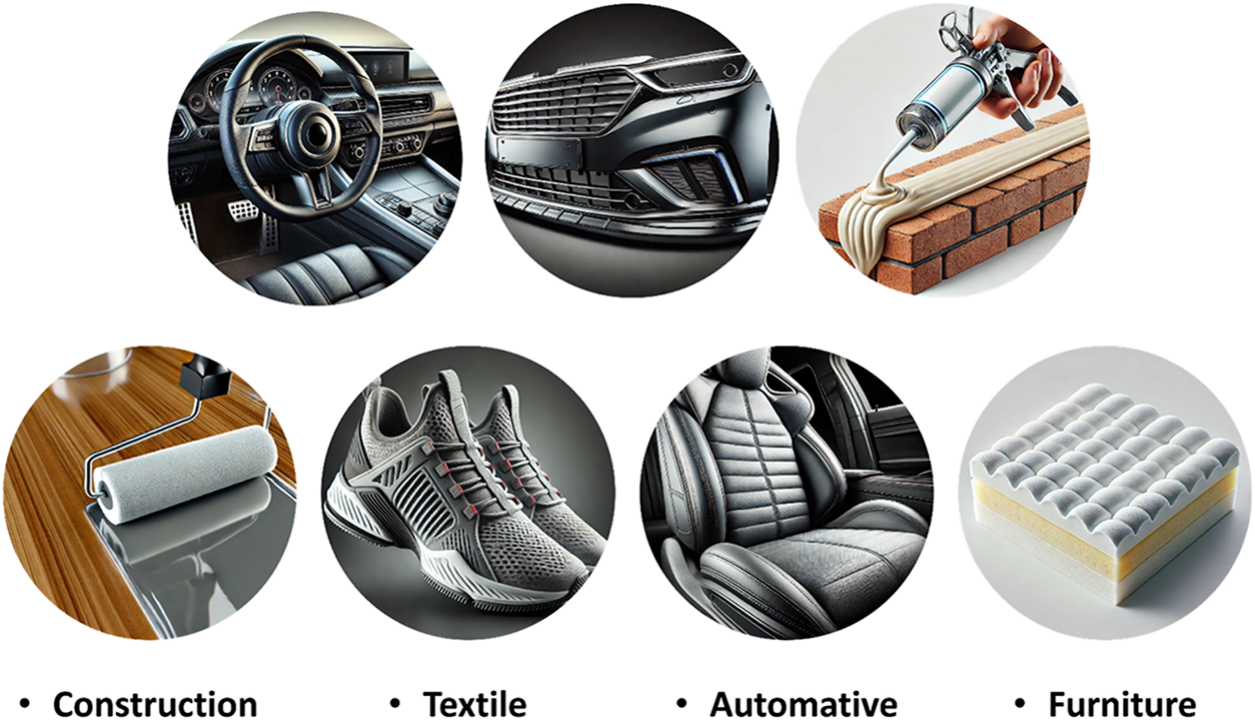
Illustration of the application areas of conventional polyurethanes (PUs). Images were generated using artificial intelligence.

PUs are essential in key industries, including construction, automative, furniture and footwear. In construction, they are widely used in rigid foam insulation panels, sealants, and adhesives, significantly contributing to energy-efficient buildings.^28^ The automotive sector relies on PUs for lightweight, durable components in both interior and exterior applications such as coatings and comfortable seat cushions.^29^ Similarly, in the furniture industry, they enhance comfort and performance in mattresses, upholstery, and protective padding. Beyond these common applications, PUs play a vital role in specialized sectors. In footwear, they provide cushioning and support in midsoles and insoles, while in textiles, they are critical for coatings and Spandex fibers. The medical field also benefits, utilizing PUs in prosthetics, wound dressings, and coatings for medical devices, addressing critical health needs.^30^ In all these examples, however, the properties of the PUs are static and cannot respond to external changes.

The traditional method for the synthesis of PUs is through the polyaddition reaction of polyfunctional isocyanates with compounds containing at least two hydroxyl groups. This reaction proceeds under mild conditions due to the high reactivity of isocyanate groups, which arises from the electrophilic nature of the carbon atom in the cumulated double-bond system of the N = C=O group.^31^ The industrial production of PUs is mainly based on aromatic diisocyanates (i.e., 4,4′-diphenylmethane diisocyanate (MDI) and 2,4-toluene diisocyanate (TDI)) (Figure 2). In addition to aromatic isocyanates, aliphatic isocyanates are also widely employed in polyurethane chemistry, particularly in light-, weather-, or heat-resistant PU coating formulations. The most common aliphatic isocyanates include 1,6-hexamethylene diisocyanate (HDI), isophorone diisocyanate (IPDI), and 4,4′-diisocyanato dicyclohexylmethane (hydrogenated MDI, H_12_MDI).^15^ There are also several aromatic and aliphatic polymeric isocyanates, which are typically used for the branched and cross-linked PUs (Figure 2).^15,18^

**Figure 2:**
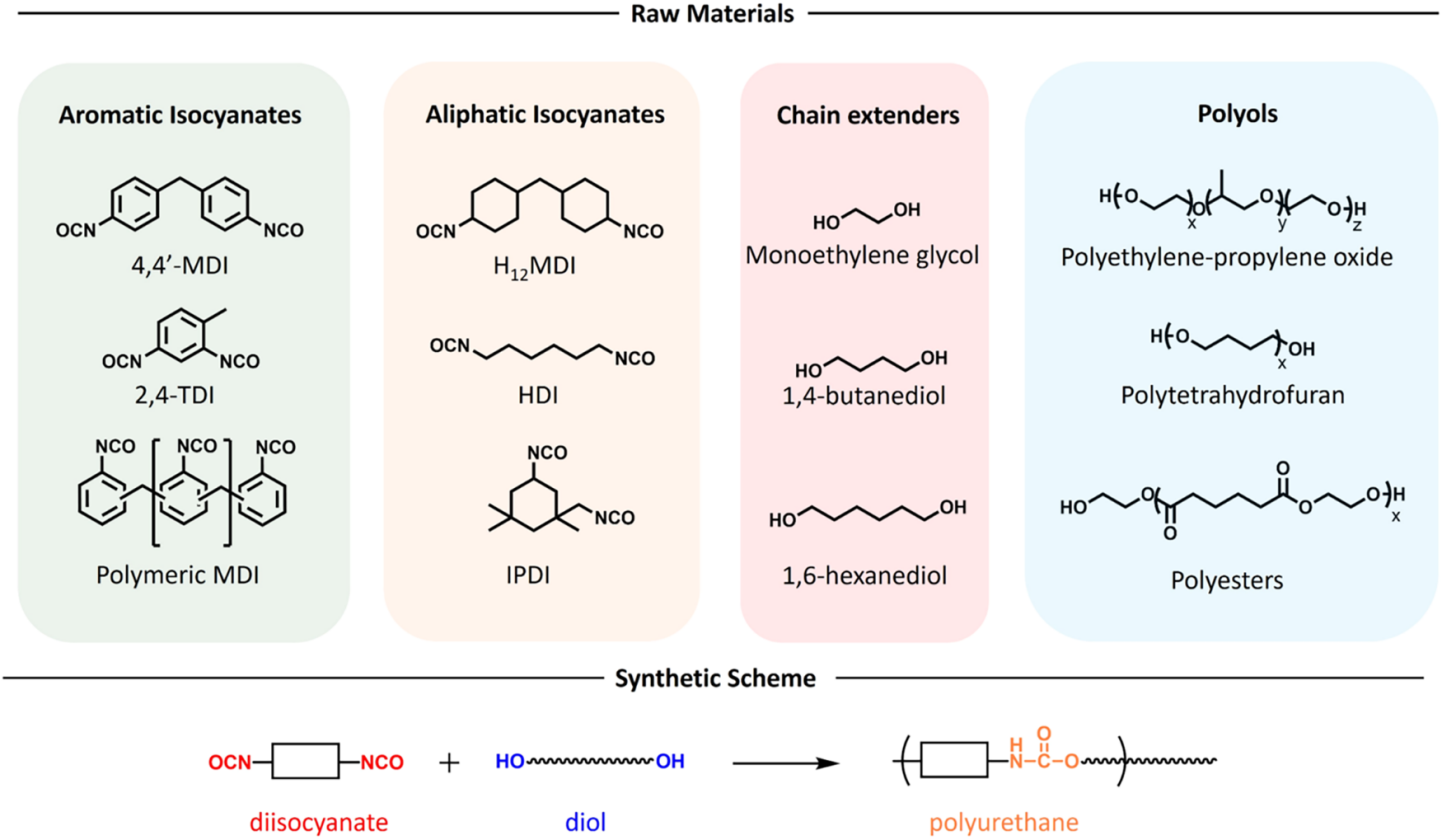
Overview of raw materials utilized in PU chemistry and general synthetic scheme for the synthesis of PUs.

Another key component involved in the synthesis of PUs is polyhydroxyl compounds, which can be used as chain extenders or polyols. Chain extenders are low-molecular-weight, well-defined diols, such as monoethylene glycol and 1,4-butanediol.^15^ Polyols, on the other hand, are macromolecular compounds with telechelic hydroxyl functional end groups. They exhibit significant diversity, with their chemical structure, molecular weight, and functionality playing a critical role in determining the final material properties.^15^ Polyols are primarily classified into two main types: poly­(ester polyols) and poly­(ether polyols). While the earliest examples in PU history employed poly­(ester polyols), today, poly­(ether polyols) account for approximately 80% of commercially used polyols.^12^ These include poly­(propylene glycol), poly­(ethylene glycol), and their copolymers, as well as polytetrahydrofurans. Poly­(ester polyols), synthesized via adipic acid condensation with diols, make up the remaining 20%. Despite offering superior mechanical strength, poly­(ester polyols) are outpaced by poly­(ether polyols) due to their lower cost and superior hydrolytic stability.^15^

The versatility of PU stems from its broad spectrum of properties, which can range from flexible to rigid and from solid to foamed materials.^15^ By careful selection of polyols, chain extenders, isocyanate precursors and the ratios between them, the properties of the PUs can be finely tailored through variations in molecular structure and morphology caused by phase separation. PU elastomers are composed of alternating soft and hard segments, offering a unique combination of flexibility and rigidity.^15^ The soft segments, derived from polyols, typically have a low glass transition temperature, which impart elastomeric properties to the polymer. In contrast, the hard segments are formed from di-isocyanates and chain extenders, containing highly polar urethane linkages. Due to hydrogen bonding among urethane groups from different chains, these segments tend to phase-separate, creating microdomains within the material. The hard segments serve as physical cross-links, and physical, mechanical, and adhesive properties of the PUs are significantly influenced by the degree of phase separation and the interconnectivity of these domains.^31^

PUs are typically processed by mixing two liquid components—polyols and isocyanates—which react to form the final material. PU processing techniques are tailored based on the specific type of PU being produced—thermoplastic or thermoset—and the intended end products.^32^ Thermoset PUs are formed using reaction injection molding, spray applications, and casting, where the two components undergo a chemical reaction to create PU materials, or are molded into solid objects. These processes result in materials that cannot be remelted after they are set. Thermoplastic polyurethanes (TPUs), on the other hand, can be processed using solvent-based methods, which involve dissolving the material in good solvents and casting, or melt-processing techniques such as extrusion, injection molding, and blow molding.^32^ The melt-processing methods create sheets, films, or intricate shapes, allowing TPUs to be softened and hardened repeatedly, enhancing their versatility for various applications. Furthermore, due to their melt-processability with excellent flexibility, durability and abrasion resistance, TPUs can also be used in the 3D printing technologies of multijet fusion, selective laser sintering and fused deposition modeling.^33^

Sustainability is an important research topic in industrial polymers. Scientific and industrial research is now devoted to designing and synthesizing PUs that are circular and biobased.^34–37^ Closed loop chemical recycling is ideal as it preserves the material’s functional properties.^38^ Mechanical recycling often results in material degradation over time, leading to downcycling due to continual backbone degradation and loss of material value.^39^ Solvent-based recycling is an alternative method for recycling TPUs: however, it requires organic solvents such as dimethylformamide and *N*-methylpyrrolidone for their solubilization and the environmental unfriendliness and hazard potential of these solvents diminish the sustainability of the process.^40–42^ To address these recycling limitations, developing PUs with built-in cleavable linkages such as acetal, imine and triazine enabling controlled depolymerization at the end of their life cycle is considered.^43–50^

## 3 Liquid Crystal Elastomers (LCEs) in a Nutshell

LCs present a unique state of matter intermediate to the solid crystalline and isotropic liquid phases, combining the fluidity of liquids with the long-range molecular order of crystals. LCs are typically composed of rigid, anisotropic mesogenic units, such as rod-like or disc-like molecules, that exhibit partial or positional order while retaining fluid-like mobility. This intermediate order gives rise to a variety of thermotropic mesophases, including the nematic, smectic, and cholesteric phases, each characterized by different degrees and types of molecular organization. The nematic phase, for instance, maintains long-range orientational order of the mesogens, whereas smectic phases display both orientational and layer positional order, and cholesteric phases introduce helical twisting of the nematic director, resulting in periodic structures with selective reflection and structural color properties. This structural anisotropy enables LCs to respond dynamically and predictably to external stimuli, making LCs essential in a wide range of applications, from display technologies to advanced optical devices and sensors. However, small-molecule LCs often lack mechanical robustness for durable use. By embedding mesogenic units either along or as side-chains of the polymer backbone, the resulting liquid crystalline polymers (LCPs) integrate the dynamic ordered nature of LCs with the structural integrity and processability of polymeric networks. Introducing a lightly cross-linked network to an LCP yields an LCE which combines the anisotropic ordering of LCs with the elastic deformability of rubbers, capable of undergoing large and reversible deformation upon triggering with heat, light, or electricity (Figure 3).

**Figure 3:**
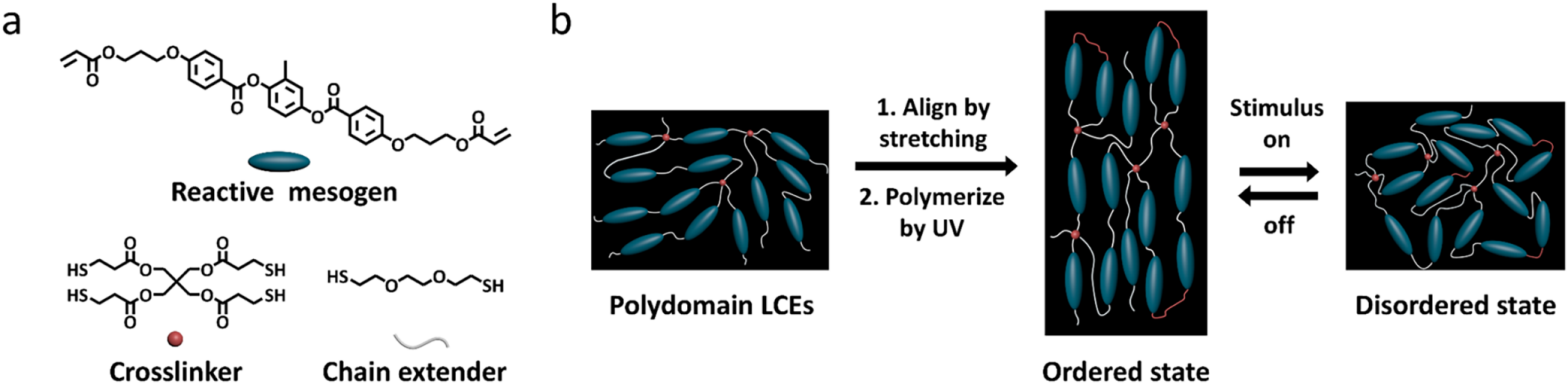
Overview of the liquid crystal elastomers (LCEs). (a) Three key material elements to synthesize common LCEs, including reactive mesogens, cross-linkers, and chain extenders. (b) Schematic drawing of the two-step alignment method for LCEs and their stimuli response. The black frames represent the macroscopic shape of the LCE at each stage.

Classical LCEs are chemically cross-linked networks: essentially thermoset polymers, where permanent covalent bonds serve as cross-links between polymer chains. They are typically prepared using reactive mesogens (RMs) through thiol–acrylate reactions, where RMs are oligomerized, followed by mechanically aligning the prepolymer (usually by stretching) to induce alignment, and then cross-linking the network via photopolymerization to lock in the orientation, resulting in a monodomain LCE with uniform alignment (Figure 3b). LC mesogens within the loosely cross-linked aligned LCE become disordered under stimuli, leading to a contraction along the alignment director and expansion perpendicular when passing by the (liquid crystalline) nematic-to-isotropic (liquid) temperature (*T*_NI_). By appropriate materials design, diverse shape transformations can be obtained in response to various stimuli, including temperature,^51^ light,^52^ humidity,^53^ magnetic^54^ or electric fields,^55^ and chemical exposure.^56^

LCEs^2–5^ and their composites^57^ have been extensively reviewed in the literature. For illustrative purposes, we present here a few example devices to give a glimpse into the wide variety of options available for actuation triggers (including light, temperature, and humidity) and for type of response (such as shrinking, bending, twisting, and color changes). First, LCE fibers can exhibit reversible contraction triggered by Joule heating from a conductive liquid metal shell (Figure 4a).^58^ Light is another popular stimulus as it can realize on-demand and remote control via adjusting light intensity and spot size. Figure 4b demonstrates a self-oscillating LCE fiber reconfiguring from a straight to helically coiled shape while lifting a screw under near-infrared light.^59^ More shape transformations can be realized by manually aligning LCE at specified angles, thereby achieving twisting or curling motions.^60^ Other LC alignments generated by printing^61^ or photoalignment^62^ are often used for RMs with low viscosity and become significantly more challenging once the LC network forms.

**Figure 4:**
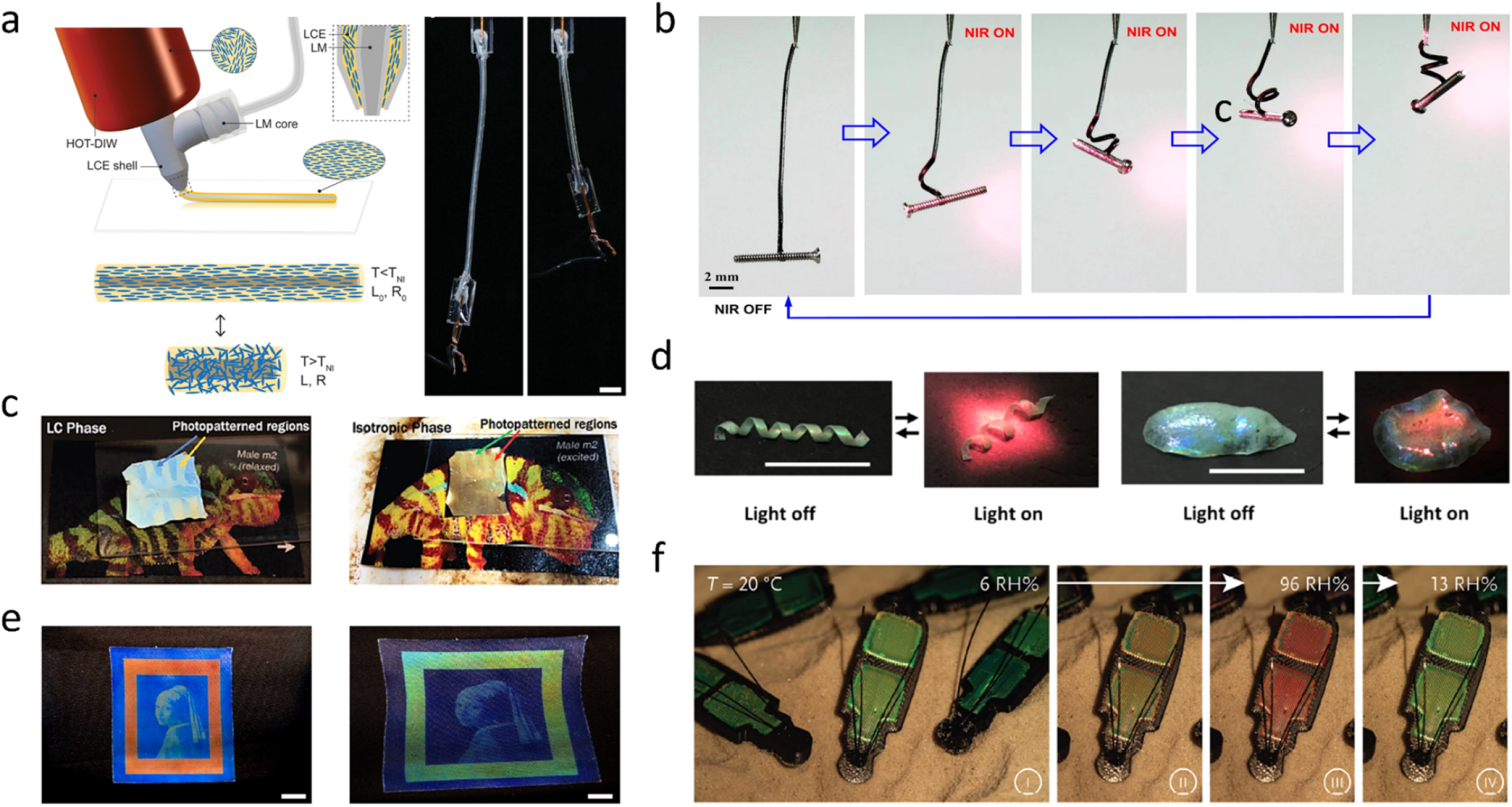
Representative shape and color changes in various LCE structures. (a) Shrinking/expanding actuation of a core–shell innervated LCE actuator under electric fields. Scale bar, 5 mm. Reproduced with permission from ref 58. Copyright 2021 John Wiley and Sons. (b) Self-winding actuation of an LCE fiber from a straight to helically coiled shape under light stimulus. Reproduced from ref 59. Available under a CC-BY license. Copyright 2021 Zhiming Hu, Yunlong Li, Jiu-an Lv. (c) Photo patterned chameleon skin in the LC and isotropic phases. Reproduced with permission from ref 63. Copyright 2020 John Wiley and Sons. (d) Simultaneous shape and color changes in a 3D spiral-shaped and cone-shaped CLCE under light stimulus. Scale bars, 10 mm. Reproduced from ref 64. Copyright 2022 American Chemical Society. (e) Mechano-chromic behavior of the CLCE imprinted with the portrait of Girl with a Pearl Earring by Johannes Vermeer. Reproduced from ref 65. Available under a CC-BY license. Copyright 2024 Albert P. H. J. Schenning, Osvaldo N. Oliveira, Johan Lub et al. (f) Gradual color changes of a 3D-printed water-responsive beetle at different relative humidities. Reproduced with permission from ref 66. Copyright 2022 Michael G. Debije, Albert P. H. J. Schenning, Luc G. Smits et al.

Color changes have also been widely investigated in LCEs. By introducing a chiral dopant, a ‘twist’ in the alignment of adjacent LC planes is generated, resulting in a helical-type structure which reflects specific wavelengths of incident light with polarization corresponding to the handedness of the cholesteric liquid crystal elastomer (CLCE). Structural color changes can be triggered by various stimuli, including temperature, mechanical deformation, humidity, and light. For example, a CLCE was designed to mimic the adaptive optical behavior of chameleon skin (Figure 4c) with simultaneous, reversible shape and blue shift color change upon heating.^63^ Inspired by the camouflage mechanisms of cuttlefish, a light-responsive CLCE actuator has been developed demonstrating both color and shape changes under illumination (Figure 4d).^64^ Mechanochromic CLCEs imprinted with high spatial resolution respond to mechanical strain, as indicated by a reversible blue shift in reflected colors upon stretching (Figure 4e).^65^ A CLCE beetle structure displays vibrant color shifts under varying humidity (Figure 4f), highlighting the potential for applications ranging from environmental sensors to adaptive optics.^66^

These examples suggest LCEs could be applied as soft actuators and sensors. However, despite a long history and unique adaptive properties, the industrial impact of LCEs remains limited^67,68^ and their commercial impact has been miniscule. One bottleneck to application of LCEs is processing: almost all monomers use acrylate end groups. Not only is it challenging to prepare complex shapes as photopolymerization is required after each alignment step, there are also no options for preparing thick films or large objects due to the restricted penetration depth of light. In addition, owing to the permanent nature of the covalent cross-links, thermoset LCEs are inherently nonrecyclable and cannot be reconfigured. One possible strategy to address these limitations can be the incorporation of dynamic covalent chemistries to produce LCE vitrimers allowing reprocessing, reshaping, and recycling of the thermoset networks. LCE vitrimers are a relatively new class of polymer network that lies between thermosets and thermoplastics. They are covalently cross-linked like thermosets but contain dynamic bonds that can exchange and reconfigure under specific conditions (typically heat and/or a catalyst), allowing the network to flow or relax stress without losing cross-link density. Another major bottleneck to widespread deployment is RMs are relatively expensive (€1000–2000/kilogram). As a result of complicated processing and higher costs, LCEs have been restricted to academic examples of small, generally flat, thin, freestanding films presented as artificial muscles, actuators, and soft robots, applications where energy efficiency and durability are enormous challenges. The building blocks, requirement of photopolymerization, and alignment techniques necessary are generally incompatible with common industrial polymer processing methods, including injection molding and hot embossing, marginalizing the societal and industrial impact of LCEs.

## 4 Perspective and Outlook

### 4.1 Current PU-LCEs Design

We believe it is well past time for the introduction of new, revolutionary polymers capable of responding to changes in their environment. In this Perspective, we propose to meet this goal by combining the responsivity of LCEs with the processability and desirable physical properties of PUs which are extremely versatile and may be made into flexible and rigid foams, fibers, elastomers, and surface coatings. Broadly, recent work classifies PU-LCEs into four categories based on the network structure and processing characteristics: thermosets, interpenetrating networks (IPNs), vitrimers, and thermoplasts.

Thermoset PU-LCEs are often synthesized by polymerizing multifunctional LC PU precursors or by introducing tri- or tetra-functional cross-linkers into LC PU chains to “set” the network.^69–72^ For example, side-chain LC PU precursors were prepared with mesogenic pendants and then cross-linked with a tetra-functional agent to form uniform PU-LCE networks (Figure 5a).^70^ The resulting thermoset networks displayed well-defined nematic and smectic LC phases and enabled multishape memory behavior that could fix and release shapes in a stepwise manner. However, thermoset PU-LCs often suffer inherent limitations in processability and sustainability. Like conventional thermosets, once polymerized, the covalent network cannot be melted or remolded, which complicates manufacturing and recycling. Shaping these materials typically requires casting or molding during synthesis, and any postfabrication reconfiguration is limited to shape memory programming. IPNs represent a strategy to combine two different polymer networks in an intertwined but noncovalently bonded nature. In PU-LCE IPNs, an LCE (or PU) network coexists with another polymer network (often a more rigid or cross-linked PU network), forming a bicontinuous blend which may mitigate the weaknesses of each component.^73–78^ The two networks are generally polymerized either simultaneously or sequentially such that they interlace without a formal chemical linkage between them. A notable example addressed the modest force output in LCEs by creating an IPN of an LC PU elastomer and a thermoset LC polyacrylate (Figure 5b).^73^ The resulting material exhibited two-way shape memory behavior, reversibly contracting and expanding up to 86% upon heating and cooling, but with an ultrastrong actuation force far exceeding that of a conventional single-network LCE. However, IPNs often inherit many of the same processing and environmental limitations as thermosets. Because at least one component network (and frequently, both) is permanently cross-linked, IPNs are essentially nonmeltable and insoluble. The fabrication of IPNs is also more complex, as it demands carefully synchronized polymerizations or simultaneous curing of one network and chain-growth of another to achieve a homogeneous morphology.

**Figure 5:**
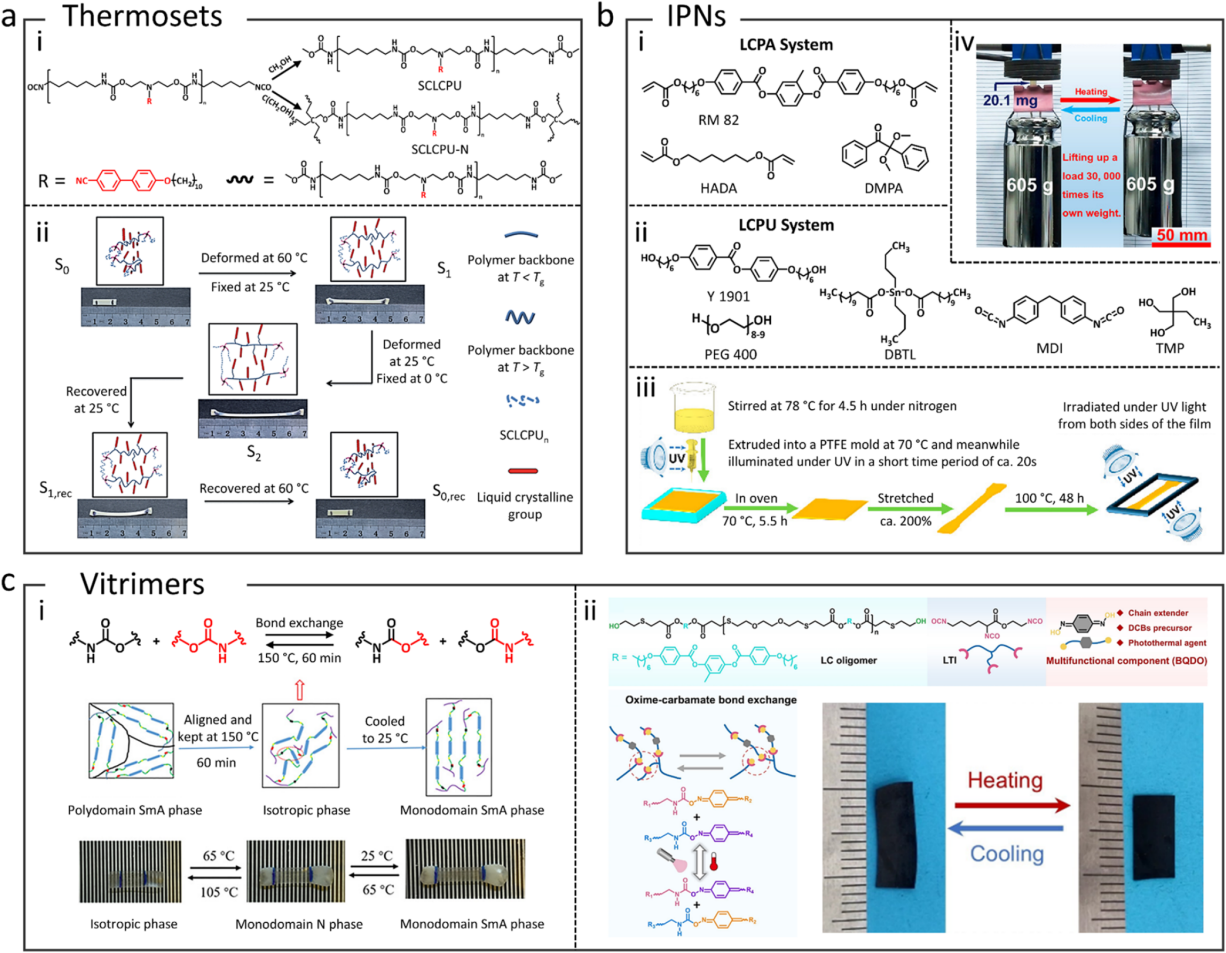
Three representative strategies for designing PU-LCEs. (a) Schematic illustration of (i) the chemical structure and synthesis of thermoset side-chain LC polyurethane. (ii) The resulting PU-LCE exhibited a triple-shape memory effect governed by the glass transition temperature of the network. Reproduced with permission from ref 70. Copyright 2013 Royal Society of Chemistry. (b) Schematic representation illustrating the formation of PU-LCE IPNs, where (i) a polyacrylate LC network and (ii) a main-chain LC PU are (iii) simultaneously polymerized. (iv) The resulting IPNs enhance the actuation capacity and enable reversible two-way thermal actuation with a maximum shrinkage ratio of 86% at 140 °C. Reproduced from ref 73. Copyright 2019 American Chemical Society. (c) Mechanism and illustration of (i) a reconfigurable vitrimer-like PU-LCE with exchangeable carbamate functional groups. Upon thermal cycling, the PU-LCE film exhibited two-stage, reversible actuation: contraction during the smectic-to-nematic transition and further shrinkage during the nematic-to-isotropic transition. Reproduced from ref 79. Copyright 2018 American Chemical Society. (ii) Chemical structures and schematic illustration of a vitrimer-like PU-LCE incorporating dynamic oxime-carbamate bonds and mechanism of the exchange reaction. Upon heating to 120 °C, the obtained aligned PU-LCE film exhibited a reversible uniaxial contraction (maximum 29%) along the mesogenic orientation. Reproduced with permission from ref 82. Copyright 2025 John Wiley and Sons.

Vitrimer-like PU-LCEs are dynamic covalent networks that retain LC order but gain the ability to be reprocessed, welded, or self-healed thanks to bond exchange reactions. Implementing vitrimer chemistry in PU-LCEs usually involves designing exchangeable linkages into the polymer backbone or cross-links. Common approaches in recent years include incorporating dynamic urethane/thiourethane bonds which undergo transcarbamoylation or dissociation-exchange at elevated temperature (Figure 5c (i)),^79,80^ or dynamic oxime-carbamate bonds (Figure 5c (ii)).^81,82^ For example, a vitrimer-like PU-LCE which can be repeatedly processed through industrial extrusion, injection molding, and hot pressing like a thermoplastic at high temperature was reported, emphasizing the recyclability and reconfigurability (Figure 6a).^80^ While vitrimer-like LC-PUs show promise, the need for bond exchange typically requires elevated temperatures or catalytic activation, which can complicate processing. For instance, the transcarbamoylation (exchange of urethane bonds) might only occur above ∼ 150–200 °C in the presence of a catalyst for an hour. This means that reprocessing a vitrimer LC-PU may require careful control to preserve LC alignment, while also avoiding thermo-oxidative degradation of the polymer structure.

**Figure 6:**
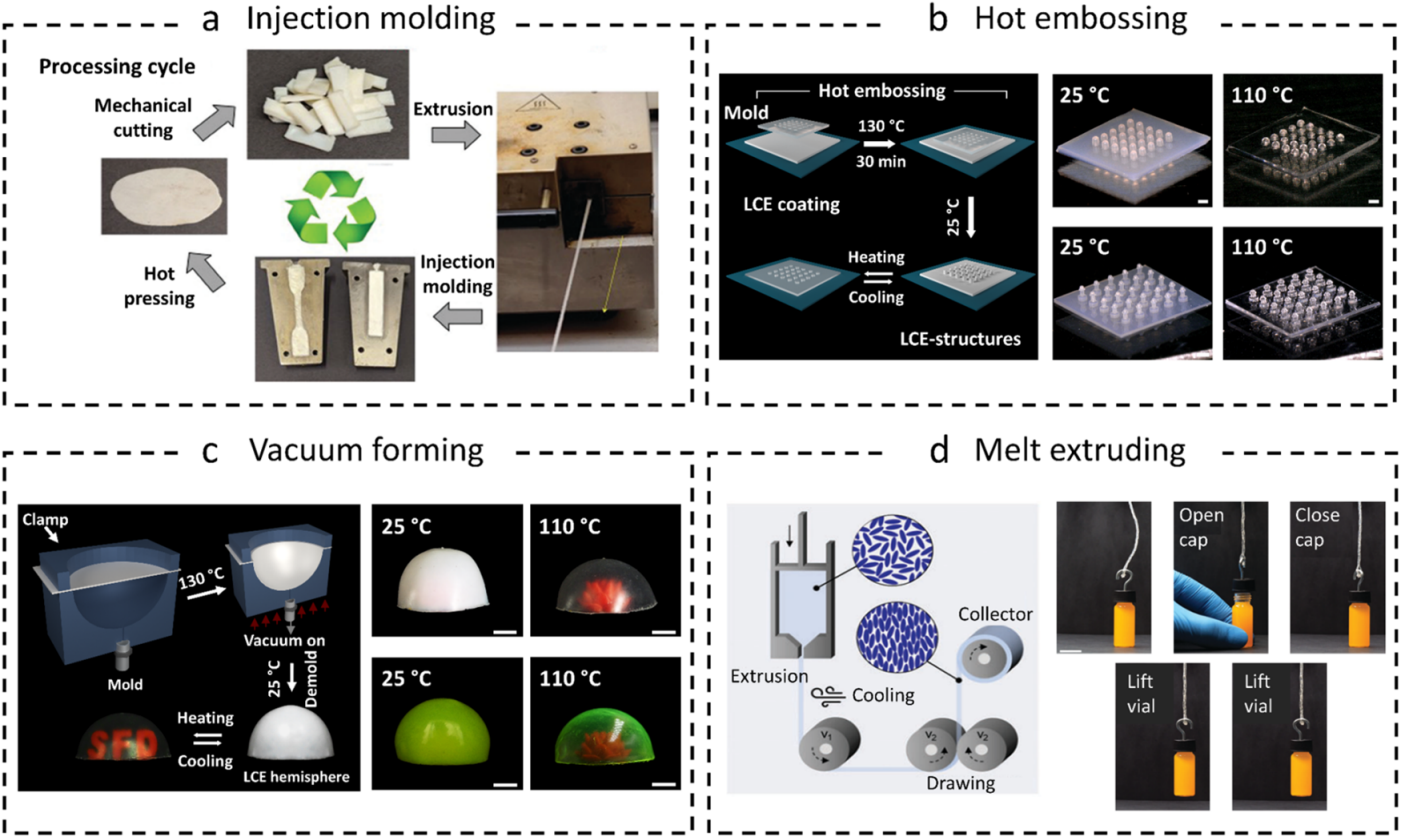
Representative examples of manufacturing PU-LCEs with common industrial technologies. (a) Schematic representation of the repeatable processing cycle postpolymerization: randomly cut PTU-LCEs are reprocessed using extrusion via a twin-screw extruder, injection molding into custom shapes, and hot pressing into homogeneous films. Reproduced with permission from ref 80. Copyright 2023 Eugene M. Terentjev, Alexandra Gablier, Mohand O. Saed. (b) Schematic of the hot embossing process (left), and temperature response of the embossed LCE surfaces (right). Scale bars, 300 μm. Reproduced from ref 89. Available under a CC-BY license. Copyright 2024 Lansong Yue, Sean J. D. Lugger, Michael G. Debije et al. (c) Schematic depicting the vacuum thermoforming process (left) and thermal actuation of 3D spherical actuators (right). Scale bars, 10 mm. Reproduced from ref 87. Available under a CC-BY license. Copyright 2024 Lansong Yue, Erik P. J. Ambergen, Sean J. D. Lugger et al. (d) Schematic melt-extrusion and drawing process of the fibers (left), and thermal actuation of the twisted fiber actuator opening and closing a screw cap and subsequently lifting the closed vial (right). Scale bar, 15 mm. Reproduced from ref 86. Available under a CC-BY license. Copyright 2023 Sean J. D. Lugger, Tom A. P. Engels, Ruth Cardinaels et al.

Thermoplastic PU-LCEs typically consist of flexible LC “soft” segment and diisocyanate-derived “hard” segments that phase-separate into microdomains. The hard domains use physical cross-links such as hydrogen bonding, giving the material elasticity and mechanical strength, yet they dissociate upon heating, allowing the PU-LCEs to be remelted and molded, and reform upon cooling without any external treatment, making them environmentally conscious, and the responsivity desired for many applications^83^ while avoiding the restrictive requirement of photopolymerization of epoxy- and acrylate-based LCEs^84^ which leads to nonreprocessable thermosets.

Truly responsive and reprocessable thermoplastic PU-LCE have not yet been reported. Initial studies into this type of material involve the simple one-pot synthesis of polythiourethane (PTU)-LCEs with “soft” LC and “hard” thiourethane segments.^85^ The PTU-LCEs have demonstrated remarkable potential for large-scale applications, including as high-performance actuators^86^ with useful optical properties^87,88^ capable of being processed by industrially relevant procedures (Figure 6). For instance, hot embossing of hydrogen-bonded PTU-LCEs has produced self-healing and reconfigurable exotic surfaces capable of reversibly responding to environmental stimuli (Figure 6b).^89^ The embossed surface structures exhibited reversible and programmable temperature and light responses, which may be erased and rewritten to alternative complex topographies by enabling multiple cycles of geometrical reconfiguration without material degradation. Other work demonstrated an integration with industrially scalable vacuum thermoforming process to create actuators with intricate 3D structures like hemispheres (Figure 6c).^87^ This enabled fabrication of 3D spherical actuators with reversible shape and transparency changes triggered by heat or light. In addition, the melt-processable PTU-LCEs have been shown to be compatible with melt extrusion, capable of producing tens of meters of fibers with excellent reprogrammability.^86,90^ Figure 6d shows that the reprogrammed three-ply twisted fibers demonstrate enhanced rotational and longitudinal forces that could open or close a vial screw cap and reversibly lift the same vial. PTUs often have comparable properties to PUs and are often easier to recycle;^91^ however, they are not always desirable because of the instability of the thiol-carbon (C–S) bond.^92^ The C–S bond in thiourethanes is inherently weaker (bond energy ∼ 218 kJ/mol) and longer (bond length ∼ 1.9 Å) than the C–O bond in urethanes which have bond energy of 330 kJ/mol and bond length of 1.4 Å due to the larger atomic size and lower electronegativity of sulfur. This weaker bond makes PTUs more susceptible to thermal degradation. Even though thermoplastic PTUs demonstrate recyclability through dynamic hydrogen bonding, repeated recycling often leads to diminished mechanical properties and actuation performance.^93^

### 4.2 Key Challenges and Emerging Strategies toward Industrial-Scale PU-LCEs

Despite recent advances, current PU-LCE systems still face constraints for practical and scalable requirements. A central issue is the continued reliance on RMs. Conventional RMs are not only expensive but often need photopolymerization for network configuration which confines fabrication to thin layers or small volumes. In addition, implementing complex director profiles in PU-LCEs is challenging due to the material’s inherently high processing viscosity. Traditional alignment techniques like magnetic or electric fields are generally only effective on low-viscosity LC monomers. Most existing LCE actuators (aligned by mechanical stretching) can only contract or bend in a single direction with small strains, whereas more complex shape changes require multiple domains or a spatial gradient in orientation. Current methods still struggle to achieve truly continuous or arbitrary director patterns in large 3D volumes. In addition, resolution and precision in programmed PU-LCE shape transformations are likewise tied to alignment limitations. For instance, direct ink writing of LCEs can achieve spatially varying alignment along the print path, but the smallest features are limited by the nozzle diameter and material flow. In contrast, photoalignment and lithographic techniques on thin LCE films can achieve much higher resolution, down to tens of microns or even smaller. However, these high-resolution patterns are generally confined to thin films. From the perspective of industrial processing, traditional PUs benefit from many standardized manufacturing techniques including melt extrusion, injection molding, blow molding, and vacuum forming, which are essential for high-throughput and cost-effective production. In contrast, current PU-LCEs are often incompatible with these techniques due to the inherently chemically cross-linked networks or requirements for mesogenic alignment.

Thus, to clear the next hurdle and enter the bulk market, there are numerous steps that must be taken. First, an appropriate PU-LCE must be synthesized. A promising approach to achieve PU-LCEs involves integrating suitable LC units into the main chain or side chain of the polyol structures (Figure 7a).^94–102^ It is crucial this integration does not compromise the liquid nature of the polyols. As the incorporation of LC components can increase the viscosity or lead to a high-melting point solid, processability and ultimate applicability becomes issues. The building blocks incorporated into the PU-LCE system should be cost-effective and their integration should be straightforward. To design and synthesize economically viable LC polyols, inexpensive and commercially available monomers could be considered as substitutes for RMs. Ester bonds in conjunction with aromatic rings are known to form rigid, rod-like structures suitable for LC cores, resembling the RM-based LCEs. One option for generating the soft LC segments of the PU-LCEs is from biobased recyclable polyols utilizing monomers derived from lignin,^103,104^ a sustainable and environmentally friendly source of aromatic phenolic compounds such as vanillin.^105,106^ A variety of inexpensive monomers bearing a strong resemblance to the chemical structure of common LCs are also commercially available. For example, a LC polyol can be synthesized by using terephthalic acid, 4-hydroxybenzoic acid, and tetraethylene glycol or low molecular weight polytetrahydrofuran (e.g., 250 or 650 g/mol). The price of these monomers is approximately €5/kg at large scales. Many derivatives of the acids are also available, allowing for a wide range of property tuning. Ideally, the synthesis of the PU-LCE would involve a one-pot synthesis process with minimal purification required, streamlining production and reducing costs while ensuring the system’s feasibility for larger-scale applications. Additionally, minimizing the use of solvents during synthesis, particularly those that are environmentally unfriendly, is another important parameter to consider to enhance sustainability and market acceptance.^41^

**Figure 7:**
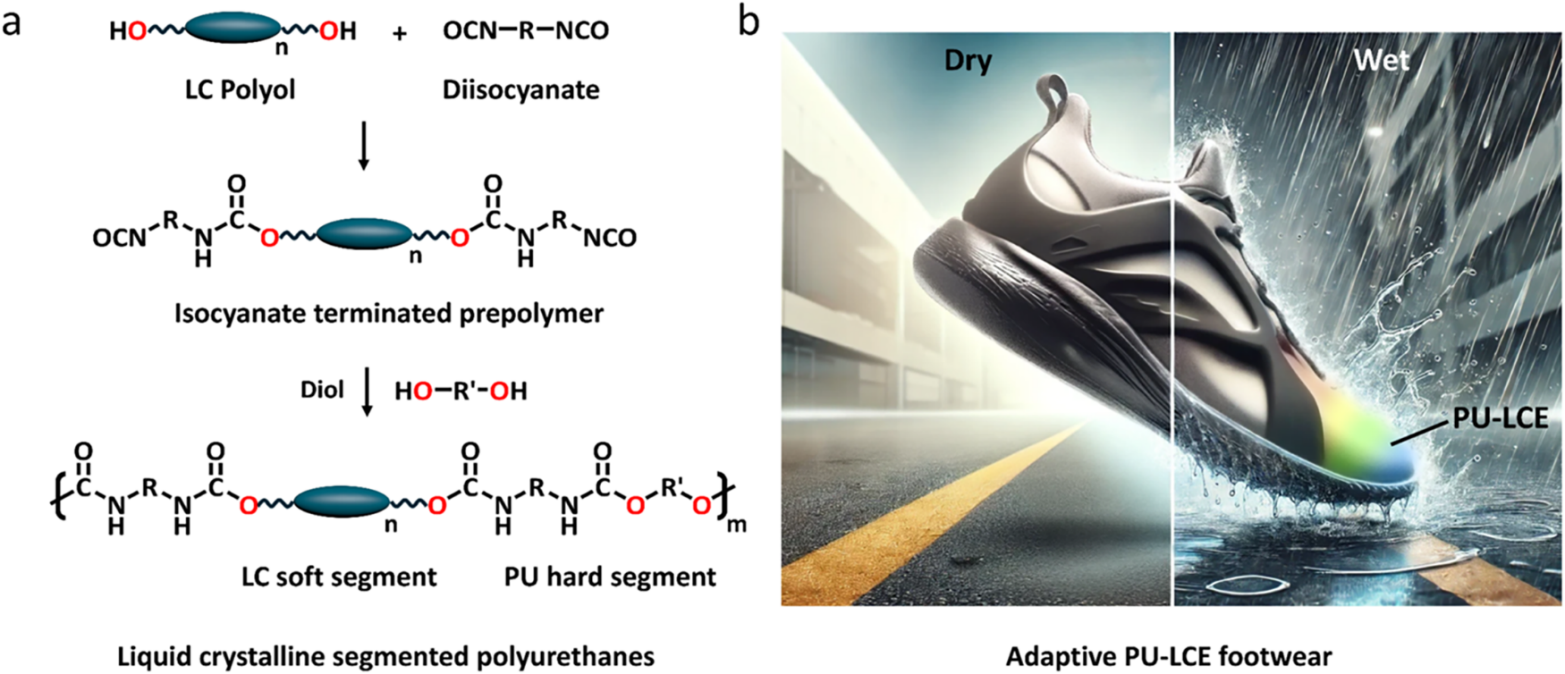
(a) Illustration of the one-pot synthesis for the preparation of PU-LCEs. (b) Illustration of adaptive PU-LCE footwear showcasing its dynamic response to environmental changes. The left figure depicts the shoe in a warm, dry state, maintaining a neutral color and standard surface properties. The right figure illustrates the transition to a cold, wet environment, where the shoe develops hydrophobic surface structures and undergoes a thermochromic shift to a rainbow color. Image was generated using artificial intelligence.

Second, addressing alignment challenges will require the high-viscosity PU-LCE can be tailored to enable mesogen orientation in flow or melt state during processing. Incorporating dynamic bonding, such as hydrogen bonding or covalent chemistries, could reduce the viscosity at moderate temperatures, allowing mesogens to reorient under applied fields or shear force before relocking upon cooling. To realize complex shape deformation behaviors beyond uniaxial contraction or bending, advances in spatial programming techniques are needed. For instance, combing scalable digital manufacturing approaches, such as direct ink writing with industrial molding or thermoforming techniques may generate high-resolution features in large-scale and achieve more complex, volumetric deformations.

To enable broader compatibility with established PU manufacturing platforms, we suggest the new PU-LCEs can be shifted from permanently cross-linked thermosets to alternative thermoplastics or vitrimers, both of which retain LC functionalities while remaining melt-processability. Thermoplastic PU-LCEs are completely reusable and reformable via the disassociation/reassociation of the physical cross-links (such as hydrogen bonds) via heat.^85–90^ These materials are ideal for continuous production lines, fiber extruding and spinning, or injection molding for wearable or consumer devices where actuation, production efficiency and recyclability are all desired. In contrast, vitrimer-like PU-LCEs behave like thermosets under normal operating conditions, maintaining structural integrity and actuation performance, but they can be reprocessed, welded, or reprogrammed under specific thermal or catalytic conditions.^107,108^ These materials can be ideal for applications such as soft robotics. However, it is important to note that one of the concerns associated with vitrimers is creep under prolonged stress, particularly at elevated temperatures. Emerging strategies that introduce closed-loop recyclable thermoset networks, for instance, via depolymerizable or cleavable cross-linkers that enable reprocessing without compromising performance may offer a path forward.^50,109^ Thus, while vitrimers expand the potentials for adaptive PU-LCEs, they may not replace thermosets. Instead, the optimal material system should be selected based on the mechanical, thermal, and functional demands of the target application.

Another key challenge for PU-LCEs is to achieve the chiral nematic (cholesteric) phase using industrial processing techniques and thus integrate the photonic structures demonstrated in regular LCEs. Incorporation of structural rather than pigmental color has advantages, including the resistance to fading and enhanced visual impact of the iridescent color. The high viscosity of PTU-LCEs has prevented formation of the cholesteric phase to date, but there are several options to finally obtaining the elusive molecular alignment. One possibility is to delay oligomerization and polymerization until the reflective bands have already been achieved.^110^ The potential of PU-LCEs to harness such structural color and dynamic adaptability is exemplified in applications like adaptive footwear, as illustrated in Figure 7b.

Circularity is another critical issue that must be taken into consideration when developing any new polymer intended to enter the market.^111^ When cleavable bonds are incorporated into the PU backbone, depolymerization can yield well-defined small molecules that are easily separated, purified, and reused for synthesis of fresh material with identical properties to the original. Such strategic design considerations will enhance the sustainability and closed-loop recyclability of PU-LCEs, making them more viable and environmentally friendly options for the market.

While we have shown examples of various processing possibilities of the future PU-LCE elastomers, other large-scale processes, such as blow and injection molding, still need to be demonstrated. The alignments so far achieved by vacuum forming and hot embossing are still limited.^87,89^ Adjusting the PU-to-LCE content, the degree of hydrogen bonding, the thickness of the films, and the possibility of prestretching to achieve higher degrees of alignment all still need to be studied.

### 4.3 PU-LCEs for Versatile Practical Applications

In this Perspective, we suggest combining liquid crystalline elastomers (LCEs) with polyurethanes (PUs) to fabricate novel responsive polymers suitable for large-scale production and industrial processing, with the potential of generating objects as shown in Figure 1: automobile seats and steering wheels, running shoes, and textiles, only now they capable of responding to their environment: as an example, automotive seats could be engineered to provide localized haptic feedback or voltage-triggered shape change, while steering wheels could actively respond to the driver’s grip or climate conditions. Additionally, PU-LCE based morphing skins could be applied to adjust the curvature of vehicle panels, where actuation could be thermally or optically triggered to adapt vehicle aerodynamics in real time by flattening or curving external contours depending on speed, direction, or wind conditions. In footwear, PU-LCEs may enable temperature or humidity-sensitive color shifts or antislip morphing patterns that adapt to wet or icy conditions, enhancing appearance and safety. In apparel, PU-LCE textile coatings or fibers could self-regulate porosity to control breathability in response to body heat or ambient humidity, offering thermoregulating comfort in sportswear and other wearable products. Wound dressings could compress upon specific light exposure and release therapeutic agents on demand by a second trigger. Obviously, application options are manyfold. All of these materials can be collected after use and reformed into new forms for new applications rather than simply being discarded as must be done with thermosets.

The approach described in this work is not restricted to only this PU-LCE combination but has the potential to be widely applicable to other thermoplastic polymers that exhibit hard and soft segments, where the soft segment is liquid crystalline. We also suggest guidelines to make LCEs less expensive and industrially processable. We hope that this Perspective will inspire scientists to develop processable responsive industrial polymers that may be produced on the large scales necessary for significant real-world impact.

## Abbreviations

PU: polyurethane
LCE: liquid crystal elastomer
LC: liquid crystal
TPU: thermoplastic polyurethane
RM: reactive mesogen
CLCE: cholesteric liquid crystal elastomer
PTU: polythiourethane
LCP: liquid crystalline polymer
IPN: interpenetrating network
